# SAFESTEREO: phase II randomized trial to compare stereotactic radiosurgery with fractionated stereotactic radiosurgery for brain metastases

**DOI:** 10.1186/s12885-023-10761-1

**Published:** 2023-03-25

**Authors:** J. A. Crouzen, A. L. Petoukhova, M. L. D. Broekman, M. Fiocco, U. J. Fisscher, J. H. Franssen, C. G. M. Gadellaa-van Hooijdonk, M. Kerkhof, M. Kiderlen, M. E. Mast, C. M. van Rij, R. Nandoe Tewarie, M. A. E. van de Sande, P. P. G. van der Toorn, R. Vlasman, M. J. Vos, N. C. M. G. van der Voort van Zyp, R. G. J. Wiggenraad, L. M. Wiltink, J. D. Zindler

**Affiliations:** 1 Haaglanden Medical Center, The Hague, The Netherlands; 2 Mathematical Institute of Leiden University, Leiden, The Netherlands; 3 Haga Hospital, The Hague, The Netherlands; 4 Zuidwest Radiotherapeutisch Instituut, Roosendaal, Vlissingen The Netherlands; 5 Erasmus MC, Rotterdam, The Netherlands; 6 Instituut Verbeeten, Tilburg, The Netherlands; 7 Catharina Hospital, Eindhoven, The Netherlands; 8 Radiotherapy Institute Friesland, Leeuwarden, The Netherlands; 9 Leiden University Medical Center, Leiden, The Netherlands; 10 Holland Proton Therapy Center, Delft, The Netherlands

**Keywords:** Stereotactic radiosurgery (SRS), Fractionated stereotactic radiosurgery (fSRS), Brain metastases, Radionecrosis, Brain necrosis, Hypofractionation, Local tumor failure

## Abstract

**Background:**

Stereotactic radiosurgery (SRS) is a frequently chosen treatment for patients with brain metastases and the number of long-term survivors is increasing. Brain necrosis (e.g. radionecrosis) is the most important long-term side effect of the treatment. Retrospective studies show a lower risk of radionecrosis and local tumor recurrence after fractionated stereotactic radiosurgery (fSRS, e.g. five fractions) compared with stereotactic radiosurgery in one or three fractions. This is especially true for patients with large brain metastases. As such, the 2022 ASTRO guideline of radiotherapy for brain metastases recommends more research to fSRS to reduce the risk of radionecrosis. This multicenter prospective randomized study aims to determine whether the incidence of adverse local events (either local failure or radionecrosis) can be reduced using fSRS versus SRS in one or three fractions in patients with brain metastases.

**Methods:**

Patients are eligible with one or more brain metastases from a solid primary tumor, age of 18 years or older, and a Karnofsky Performance Status ≥ 70. Exclusion criteria include patients with small cell lung cancer, germinoma or lymphoma, leptomeningeal metastases, a contraindication for MRI, prior inclusion in this study, prior surgery for brain metastases, prior radiotherapy for the same brain metastases (in-field re-irradiation). Participants will be randomized between SRS with a dose of 15–24 Gy in 1 or 3 fractions (standard arm) or fSRS 35 Gy in five fractions (experimental arm). The primary endpoint is the incidence of a local adverse event (local tumor failure or radionecrosis identified on MRI scans) at two years after treatment. Secondary endpoints are salvage treatment and the use of corticosteroids, bevacizumab, or antiepileptic drugs, survival, distant brain recurrences, toxicity, and quality of life.

**Discussion:**

Currently, limiting the risk of adverse events such as radionecrosis is a major challenge in the treatment of brain metastases. fSRS potentially reduces this risk of radionecrosis and local tumor failure.

**Trial registration:**

ClincalTrials.gov, trial registration number: NCT05346367, trial registration date: 26 April 2022.

## Background

In addition to surgery, targeted agents and/or immunotherapy can effectively treat brain metastases from several subtypes of cancer [1]. At the same time, stereotactic radiosurgery (SRS) is now widely available for the treatment of multiple brain metastases [2]. The advantage of SRS over whole brain radiotherapy is improved local tumor control and decreased toxicity [3]. In the current era of personalized medicine, the main question is how to integrate SRS into the multimodality treatment for brain metastases with systemic therapies and surgery. This is complex and is decided in the multidisciplinary board [4]. In the Netherlands, SRS is indicated for brain metastases smaller than 3 cm, for inoperable metastases, and when there is no need to acquire a pathologic diagnosis.

The number of long-term survivors with brain metastases has increased due to the introduction of more effective systemic therapies including immunotherapy and targeted agents [5]. The latest Graded Prognostic Assessment (GPA) model predicts a median survival of just under 4 years in the most favorable prognostic group after initial brain metastases treatment [5]. As a result, avoidance of long-term side effects is more important. Brain necrosis or radionecrosis is one of the most relevant side effects after SRS, affecting between 17 and 50% of patients, especially in the setting of immunotherapy [6–10]. Radionecrosis is a reaction of healthy brain tissue to radiotherapy characterized by necrosis and fibrinous exudate near the edge of an irradiated brain metastasis [11]. Radionecrosis may cause focal neurological deficits, neurocognitive dysfunction, and seizures, and frequently requires corticosteroids, bevacizumab, or sometimes even surgery. Furthermore, it can create anxiety in the follow-up process as it can be difficult to distinguish between radionecrosis and tumor progression. Dosimetric constraints are in place in order to prevent radionecrosis, but overly strict adherence to these constraints can negatively impact local tumor control rates [12].

Fractionated radiosurgery (fSRS) has been used to reduce the risk of side effects compared to single fraction SRS. With fSRS, the ablative dose is delivered in multiple fractions over several days, instead of delivering the ablative dose within half an hour [13]. Several retrospective studies have shown improved local control and less radionecrosis when using fSRS compared with single fraction SRS, especially in larger metastases [8, 14–19]. The improved local control is presumably due to a radiobiological advantage and a higher biologically effective dose (BED) delivered with fSRS. The 2022 ASTRO clinical practice guideline “Radiation Therapy for Brain Metastases” recommends more research to fSRS in order to reduce the risk of radionecrosis [20].

Randomized trials comparing fSRS with single fraction SRS are lacking. To achieve local tumor control rates of ≥ 90% at one year, a cumulative BED of at least 50 Gy should be delivered. This is achieved with 35 Gy in five fractions (BED is greater than 50 Gy). Another potential benefit of fSRS over single fraction SRS is better induction of the abscopal effect. This is a systemic anticancer response due to radiation-induced DNA damage. It is hypothesized that this effect is caused by the activation of a cytosolic DNA sensing pathway mediated by cyclic GMP-AMP synthase (cGAS) and stimulator of interferon genes (STING). This pathway leads to adaptive immune responses which induce cell death even outside of the targeted sites [21, 22].

To our knowledge, no prospective studies have been performed to compare fSRS with single fraction SRS in the treatment of brain metastases. In this randomized study, we aim to compare the incidence of adverse local events in patients with brain metastases treated with 1 or 3 fractions versus fSRS (5 fractions).

## Methods/design

This is a multicenter phase II prospective randomized trial with two study arms. The standard treatment is SRS in one or three fractions. The experimental arm is fSRS in five fractions.

The primary objective of this study is to compare the incidence of any local event (ALE), which is defined as either local tumor failure or radionecrosis, in the experimental arm to the incidence the standard treatment arm. We hypothesize that the experimental arm will have a lower incidence of ALE. The primary endpoint is the incidence of either local tumor failure or radionecrosis according to the Response Assessment in Neuro-Oncology Brain Metastases (RANO-BM) within two years after radiotherapy [23]. Radionecrosis is defined as progression according to nadir in combination with a functional MRI, i.e. low perfusion/low cerebral blood flow in the treated brain metastasis. The nadir is the smallest size of the brain metastases after SRS. Another option to define radionecrosis is pathological verification of the absence of vital tumor cells after resection.

The secondary objectives are to determine if fSRS provides less toxicity (CTCAE version 5.0), better overall survival, and better quality of life compared with the standard of care. The secondary endpoints are measured at predetermined time points until two years after treatment. Secondary endpoints include the use of corticosteroids, bevacizumab, antiepileptic drugs, or salvage treatment and also the incidence of distant brain recurrences. Quality of life is measured with the EORTC QLQ-BN20, EORTC QLQ-C30, EQ-5D 5L.

The study will include patients with a solid primary tumor and one or more brain metastases referred for SRS. A high resolution contrast-enhanced MRI scan is required prior to SRS. The study will take place in a clinical setting in Dutch hospitals. Inclusion criteria are age ≥ 18 years, Karnofsky Performance Status ≥ 70, and the ability to provide written informed consent. Exclusion criteria include patients with a small cell lung cancer, germinoma or lymphoma, leptomeningeal metastases, a brain metastasis with a PTV of ≥ 20 cm^3^ when metastasis is located in the brainstem, a contraindication for MRI, prior inclusion in this study, prior surgery for brain metastases, and prior radiotherapy for the same brain metastases (salvage SRS of new distant brain metastases after prior SRS on other brain metastases is allowed; in-field re-irradiation on current target brain metastases is not allowed). Patients are randomized into either standard or experimental arm in a 1:1 ratio.

The definitive number of brain metastases and the definitive maximum lesion diameter in any direction of the largest brain metastasis is determined on a gadolinium contrast-enhanced T1-sequences MRI with maximum slice thickness of 1.5 mm (field strength 1.5–3.0 Tesla with a 3D-distortion correction protocol). Systemic treatments are interrupted if necessary, according to the Dutch guidelines for brain metastases to avoid additional toxicity. Participants randomized into the standard treatment arm will receive a dose of 15–24 Gy in one fraction or 24 Gy in three fractions, depending on the brain metastases volume. Participants randomized into the experimental cohort will receive 35 Gy in five fractions, or 30 Gy in 5 fractions if the brain metastasis is located in the brainstem. Detailed dose prescription information is shown in Table 1.

**Table 1:** Dose prescription for brain metastases

PTV of brain metastases (cm3)	Dose 1 fraction (Gy)	Dose when metastasis in brainstem (Gy)	Dose 5 fractions (Gy)	Dose when metastasis in brainstem (Gy)
< 1	24	16	35	30
1–10	21	16	35	30
10–20	18	16	35	30
20–65	15 or 3 × 8	No SRS	35	No SRS

**Fig. 1:**
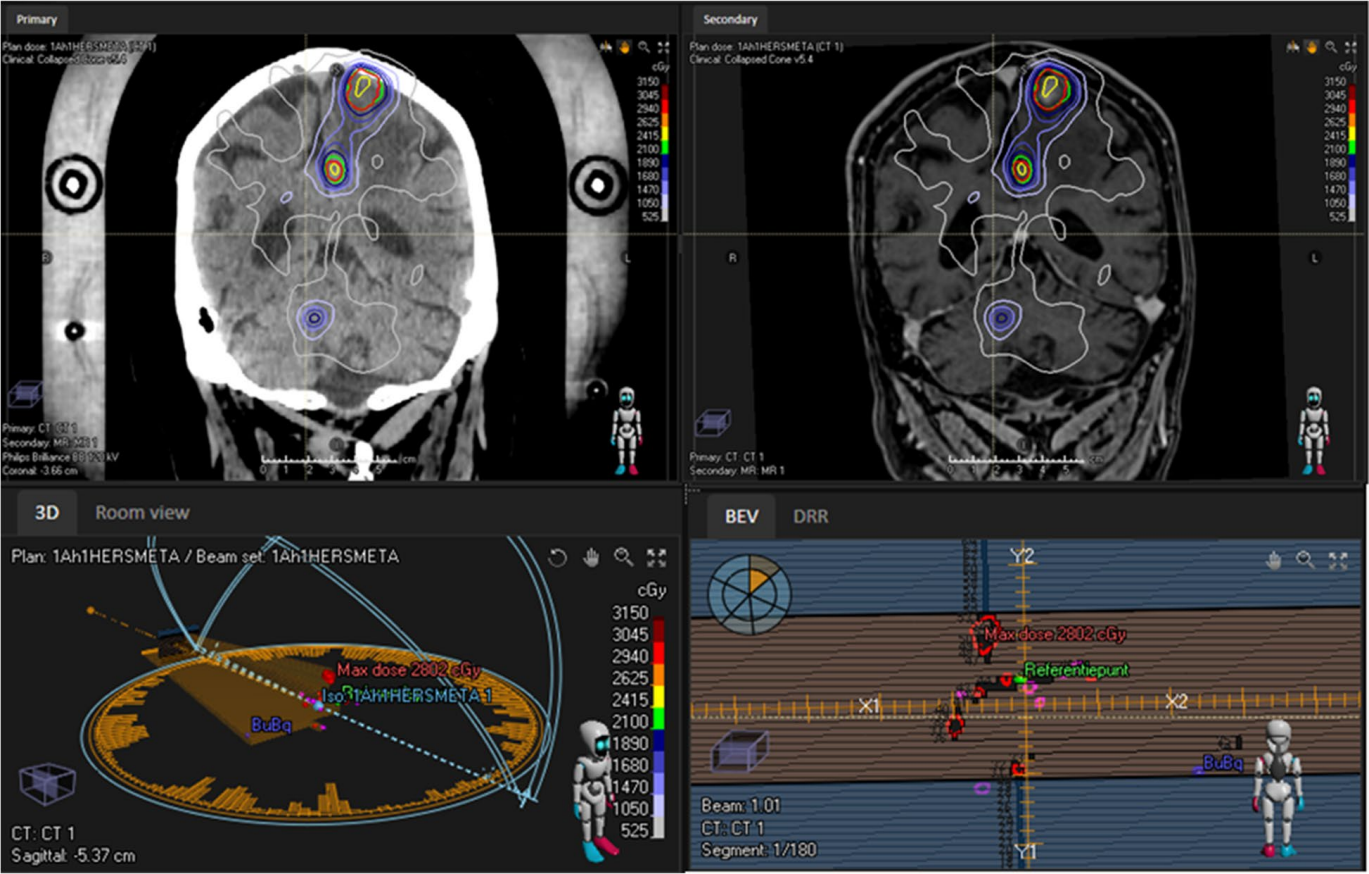
Example of treatment plan for multiple brain metastases

The gross tumor volume (GTV) is defined by contouring the contrast-enhancing border of the brain metastases on a gadolinium-enhanced T1-weighted MRI scan. Organs at risk (optic nerves and chiasm, brainstem, etc.) are contoured according to European Particle Therapy Network consensus [24]. The dose constraints for organs at risk are shown in Table 2. The planning target volume (PTV) is defined by a 0–2 mm isotropic expansion of the GTV, depending on local standards. If a brain metastasis is located inside or adjacent to the brainstem, the PTV margin will be 0 mm. 99% of the PTV needs to be covered with the prescribed dose. No minimum or maximum dose is defined, but generally speaking Dmax is approximately 140% of the prescribed dose for LINAC based SRS and approximately 200% for Gamma Knife SRS. Tumor volume and treatment plan characteristics are reported (maximum dose, prescribed dose) in Table 3. Participants will be fixed in supine position with a thermoplastic mask or stereotactic non-invasive frame, with or without a bite block or other fixation. The accuracy of the stereotactic fixation system should be sufficient so that intrafraction motion does not exceed the CTV-PTV margin. If a margin of 0 mm is used, the maximum intrafraction motion should be less than 0.5 mm, with a SD of less than 0.25 mm. A planning CT scan with ≤ 2 mm contiguous slices (preferable CT slice thickness = 1 mm) will be fused to a contrast-enhanced MRI scan. The interval between the planning MRI and actual SRS treatment is preferably 1 week, but cannot exceed 3 weeks. An example of a treatment plan is shown in Fig. 1. Tumor response evaluation as well as presence of new brain recurrences are monitored every three months after SRS using a contrast-enhanced (single—triple dose Gd is allowed) T1- sequences, field strength 1.5–3.0 Tesla, with a 3D-distortion correction protocol including perfusion MRI.

**Table 2:** Organs at risk dose constraints

	1 fraction (Gy)		3 fractions (Gy)		5 fractions (Gy)	
	Optimal	Mandatory	Optimal	Mandatory	Optimal	Mandatory
Brainstem Dmax	10	18	24	27	23	30
Cochlea Dmean	4	9	17	20	22	25
Chiasm Dmax	8	10	15	23	20	25
Lens Dmax	1	3	2	4	3	5
Optic nerves Dmax	8	10	15	23	22	25
Pituary gland Dmean	8	10	15	23	22	25

*Dmax* Maximum dose, *Dmean* Mean dose

**Table 3:** Acceptable and unacceptable variation in dose

	Per protocol dose	Acceptable variation	Unacceptable variation
PTV to be covered	99%	98% < V100% < 99%	V100% < 98%
Dmax OAR if volume of OAR < 2 cm3	0.035 cm3	D0.035cm3 ≤ Dmax	D0.035cm3 > Dmax
Dmax OAR if volume of OAR ≥ 2 cm3	2%	D2% ≤ Dmax	D2% > Dmax

*PTV* Planning target volume, *Dmax* Maximum dose, *OAR* Organ at risk

In the Netherlands, all treatment centers have state of the art hardware to deliver SRS. Therefore, LINAC based SRS, CyberKnife SRS, and GammaKnife based SRS are allowed in this study. Comparison (benchmarking) of treatment plan quality of SRS will be done within this study to further optimize the SRS technique of the participating centers.

The voluntary questionnaires regarding quality of life are sent to participants prior to radiotherapy (baseline measurement), and then at 3, 6, 12, 18, and 24 months after treatment. Toxicity according to CTCAE v5.0 (including fatigue, cognitive disturbance, alopecia), the use of corticosteroids, bevacizumab, and antiepileptic drugs are evaluated every 3 months up to two years.

Differences in the composite endpoint (either radionecrosis or local tumor failure) at two years after radiotherapy are calculated as a percentage within each study arm. The binomial test will be used to compare the percentage between the two cohorts. Summary tables for continuous variables will include mean and standard deviation. Summary tables for categorical variables will include number (N) and proportion. If the data is normally distributed, the means will be compared using independent samples Student’s T-Tests. In case of violation of the normality assumptions, non-parametric tests will be used. Proportions will be compared by using Chi-square testing. Unless otherwise indicated, tests will be 2-sided. Secondary study parameters will be presented per cohort. Due to the presence of repeated measurements, mixed modelling will be used to investigate the effect of fSRS on quality of life and epilepsy. Overall survival (from first day of radiotherapy treatment until death) will be estimated by using Kaplan–Meier methodology. To assess whether there is difference between survival in the cohorts, the Log-rank test will be used. To investigate the effect of prognostic factors on survival, a Cox regression model will be used. To estimate the cumulative incidence of adverse local events (ALE), a competing risk model with death as a competing risk will be estimated [25]. Fine and Gray’s test will be employed to assess the difference between cumulative incidence in the two cohorts [26]. Cause specific hazard Cox model will be employed to investigate the effect of prognostic factors on the cumulative incidence of ALE. To account for non-compliance and protocol deviation, the analysis will be performed based on the intention to treat concept. All analyses concerning the competing risk model will be performed in R environment by using the library mstate and cprisk [27].

The number of participants required for the study was determined by a power calculation with PASS® software (NCSS Statistical Software). The power calculation was performed for a comparison of means with two-sided alpha 0.05 and power of 0.80. This leads to a sample size per group of 59 participants. To account for drop out, the sample size was increased by 10%, so the total number of participants required is 130.

Data will be anonymized and will be entered into an electronic data capture system. Randomization is performed by a validated variable block randomization model within the data capture system. This randomization algorithm is constructed to divide randomized inclusions across groups in variable block sizes to ensure true randomness during the allocation. There will be stratification by center. Before randomization, any participant will be replaced if they are withdrawn for any reason. These patients are not included in the statistical analysis. After the randomization, patients withdrawing for any reason will not be substituted by additional patients and these patients are analyzed by intention to treat.

## Discussion

Retrospective studies have shown a potential benefit in the incidence of local tumor failure and radionecrosis of fSRS in the treatment of patients with brain metastases. However, prospective randomized studies are necessary to confirm these results. The primary endpoint of this trial is progression after (f)SRS according to RANO on a T1gd contrast MR. Progression can be either tumor recurrence or radionecrosis, or a mixture of both options, which is often hard to differentiate in clinical practice. Therefore, this composite primary endpoint is used.

## Data Availability

Not applicable, as data collection and analysis has not yet taken place. Anonymized data will be stored in an approved and secure cloud-based clinical data management platform. All principal investigators will be given access to the cleaned data sets of their own treatment site and will be given access to data sets of other sites by request. Results will be published unreservedly regardless of their nature.

## Abbreviations

ALE: Adverse local events
ASTRO: American Society for Radiation Oncology
BED: Biologically effective dose
cGAS: cyclic GMP-AMP synthase
CT: Computed tomography
CTCAE: Common Terminology Criteria for Adverse Events
CTV: Clinical target volume
fSRS: Hypofractionated stereotactic radiosurgery
Gd: Gadolinium
GPA: Graded Prognostic Assessment
GTV: Gross tumor volume
MRI: Magnetic resonance imaging
PTV: Planning target volume
RANO-BM: Response Assessment in Neuro-Oncology Brain Metastases
SD: Standard deviation
SRS: Stereotactic radiosurgery
STING: Stimulator of interferon genes
WBRT: Whole brain radiotherapy

## Declarations

### Content for publication

Not applicable. This manuscript contains no individual person’s data. If in future publications an individual’s data is used, consent for publication will be obtained from that person.
